# Young lung cancer: from diagnosis to survivorship

**DOI:** 10.3389/fonc.2025.1570143

**Published:** 2025-06-26

**Authors:** Narjust Florez, Lauren Kiel, Rebekah Kaufman, Jaclyn LoPiccolo, Biagio Ricciuti, Angela Morabito, Olayinka Fakorede, Courtney Mantz, Coral Olazagasti, Nishwant Swami, Duaa Kanan, Laura Alder, Arthi Sridhar, Cristiane Decat Bergerot, Bianca Bye, Ana I. Velazquez, Alice T. Shaw

**Affiliations:** ^1^ Lowe Center for Thoracic Oncology, Dana-Farber Cancer Institute, Boston, MA, United States; ^2^ Harvard Medical School, Boston, MA, United States; ^3^ Sylvester Comprehensive Cancer Center, University of Miami Miller School of Medicine, Miami, FL, United States; ^4^ Department of Medicine, Hospital of the University of Pennsylvania, Philadelphia, PA, United States; ^5^ University of Illinois College of Medicine Peoria, Peoria, IL, United States; ^6^ Duke Cancer Center, Duke University School of Medicine, Durham, NC, United States; ^7^ Division of Hematology and Oncology, UT Southwestern Medical Center, Dallas, TX, United States; ^8^ Centro de Câncer de Brasília, Instituto Unity de Ensino e Pesquisa, Grupo Oncoclinicas, Brasília, Brazil; ^9^ Young Lung Cancer Initiative, Raleigh, NC, United States; ^10^ Department of Medicine, University of California San Francisco, San Francisco, CA, United States

**Keywords:** young patients, lung cancer, psychosocial, targeted therapy, diagnostic delays

## Abstract

**Background:**

Young patients with lung cancer represent a distinct population, with unique disease and treatment-related characteristics, as well as psychosocial and survivorship needs. Nevertheless, this population remains vastly understudied.

**Methods:**

We review the unique clinicopathological characteristics and needs of young patients with lung cancer, including topics such as incidence rates, diagnostic challenges, genomics, treatment patterns and outcomes, psychosocial needs, fertility and sexual health, and palliative care. We discuss emerging and understudied data, provide recommendations on aspects in which future research is warranted, and advocate for actionable strategies that multi-disciplinary healthcare teams may adopt to provide more personalized and equitable care.

**Results:**

Though epidemiological trends suggest an overall decrease in lung cancer incidence among all age groups, recent increasing incidences have been reported among certain young populations in the U.S., as well as among Hispanic women and women in certain European countries. Young patients are significantly more likely to be female or Asian/Pacific Islander, have no tobacco use history, metastasis to the brain, and a higher frequency of somatic mutations or rearrangements. Diagnostic delays pose a considerable concern to young patients with lung cancer and may contribute to how these patients are more likely to be diagnosed with advanced disease than their older counterparts. However, young patients demonstrate improved survival compared to older patients, underscoring the importance of survivorship care. Young patients are more likely to be diagnosed at a disruptive time in their lives, rendering them with distinct psychosocial needs and financial toxicity. Future data on treatment-related effects on fertility and sexual health for young patients is warranted, as is the data related to complementary medicine use. Training in palliative care and promoting a positive attitude towards supportive care is also essential.

**Conclusions:**

Young patients with lung cancer represent a distinct patient population, necessitating disease management that is markedly different from that of older patients with lung cancer. Future research, some of which are highlighted by this Review, will aid in elucidating risk factors, survival rates, and clinical, genomic, and histopathological characteristics of young-onset lung cancer to improve screening, early detection, prevention, and treatment of this understudied population.

## 1 Introduction: incidence and etiology of young-onset lung cancer

The incidence of lung cancer in patients under 50 years of age, most of whom have no history of tobacco exposure, is alarming and demands urgent attention by the medical and research community. This review aims to reduce the educational gap in appropriate care delivery for this understudied population by providing a comprehensive analysis of the unique clinicopathological characteristics and needs of young patients with lung cancer from diagnosis to end-of-life care.

Lung cancer remains the leading cause of cancer death worldwide (1). While the incidence of lung cancer has declined from 2012–2021 by 3% per year in men and 1.4% per year in women (2), more people still die from lung cancer annually than colon, breast, and prostate cancers combined (3). In recent years, there has been growing attention on young-onset lung cancer; however, a definitive definition of “young patients” has yet to be established. Proposed age cutoffs include 40, 45, or 50 years, therefore, the age ranges from primary data reported in studies included in this review vary by context.

Data on incidence trends regarding young-onset lung cancer remains an area of active investigation. In recent years, an increasing incidence of lung cancer among patients <50 years old has been reported among Hispanic women (4) and women in France, Italy, and Spain (5); increasing or stable incidence rates have also been reported in China (6) and Hong Kong (7). It is unclear if the incidence of lung cancer in younger patients is increasing globally, however, as most recent data suggests a decrease in lung cancer incidence in all age groups, including in countries such as the United States (U.S.) (8–10). Contemporary, expanded studies in younger age groups, incorporating both histology and race/ethnicity, are needed to definitively answer this question. In China, the age standardized incidence rate (ASIR) per 100,000 individuals was 7.38 in 2019, higher than the 5.30 ASIR in 1990 (6). In the U.S., according to Surveillance, Epidemiology, and End Results (SEER) data from 2017-2021 (11), 0.2% of new lung cancer cases were among 20–34-year-olds, 0.9% in 35-44-year olds, and 21.8% in 45-54-year-olds. Despite overall decreasing incidence in younger populations, slightly increasing incidences were observed for certain populations under 50 years including Non-Hispanic Asian/Pacific Islander females, Non-Hispanic Black men, and Black (including Hispanic) men. Between 1-10% of patients with lung cancer in Latin America (12) are younger than 40, though the incidence of genomic driver mutations vary across countries. For instance, Northern Latin America tends to have higher incidences of EGFR mutations and ALK alterations than the southern part of the region.

Contrary to popular belief, a recent retrospective Chinese review (13) of 82 patients with lung cancer <35-years-old found that nearly all (98.6%) had no family history of lung cancer nor personal history of tobacco use (14) (71.6%). This relative lack of tobacco use among young patients, particularly women, has garnered much attention. Since 2018, higher lung cancer incidence rates that are irrespective of tobacco use have been reported among females aged 35–54 years in the U.S., reversing historically higher rates in men (4). A recent supplementary study (15) found that lung cancer incidence rates in women were equal to or higher than rates in their male counterparts in 40 of 51 states, with statistically significant differences in 20 states; the highest female-to-male incident rate ratios found in North Dakota (1.53), Idaho (1.37), and Wyoming (1.35). Notably, current and ever smoking prevalence in women compared to men was statistically significantly lower or similar in 33 and 34 states, respectively, supporting how higher incidence rates in young women are unexplained by differences in smoking prevalence. However, the impact of second-hand smoke, believed to increase the risk of lung cancer by 24% in non-tobacco users (16), warrants further analysis. With a recent report from the American Cancer Society depicting how women under 50 years old are 82% more likely to develop any cancer than their male counterparts (2), factors unique to women, such as the impact of childbirth and oral contraceptive use (17), are also ripe areas for exploration. Some have further hypothesized that risk factors such as radon gas exposure and air pollution may be contributing to young-onset cases (18, 19), similar to the environmental exposures that may be contributing to the rise in early-onset colorectal cancer cases (20). The observation that younger patients often lack tobacco or identifiable environmental exposure additionally suggests that germline predisposition may contribute to lung cancer development in young people, which is an ongoing area of investigation that is challenging due to the large numbers of individuals required to identify shared but rare genetic changes.

## 2 Diagnostic challenges

Although the survival rate for metastatic lung cancer has improved over the past four decades, the 5-year disease survival rate remains low at only 9% for non-small cell lung cancer (NSCLC) and 3% small cell lung cancer (SCLC) (21), highlighting the importance of early diagnosis. Indeed, delayed diagnosis of lung cancer poses a significant concern, particularly to young patients, as it may impact disease stage and overall survival (OS) (22). Patients may present with general pulmonary symptoms that lead to misdiagnoses (23), such as allergies, asthma, tuberculosis (24), sarcoidosis (25), viral infections, and pneumonia (26). Additionally, due to the disease’s stigma and association with tobacco use and older age, young patients and individuals with no prior tobacco use may be at greater risk of misdiagnosis due to the perceived low likelihood of developing lung cancer (27). Lung cancer screening guidelines (28) may also impact contribute to diagnostic delays, as recommendations currently only focus on individuals aged 50–80 with a history of at least 20 pack-years of tobacco use, thereby excluding young patients, even if they have extensive exposure to second-hand smoke or other risk factors.

Gender bias may also contribute to diagnostic delays, as women experience significantly longer times to diagnoses than men (29, 30). At least 33% of women newly diagnosed with lung cancer have three or more visits with their general practitioner before being referred for further imaging or biopsy, compared to only 3% of patients with breast cancer (31). Women are also 32% less likely than men to report a lung cancer screening discussion with a primary care provider and 32% less likely than men to be aware of available lung cancer screening tests (32). Further, women are more likely to have additional follow-up visits and a greater number of consultations rather than an immediate diagnostic intervention that could lead to a timely diagnosis (33, 34). Gendered association of certain risk factors such as tobacco (35) may also contribute to delays, as women with a history of tobacco use are *still* more likely to have a late-stage diagnosis due to delays faced in obtaining appropriate imaging and tissue diagnosis (36).

Diagnostic delays may be one contributing factor to how young patients are more likely than their older counterparts to be diagnosed with later-stage or advanced disease (13, 37–40), with a larger number having node-positive disease (60% vs. 51%) (41). Unsurprisingly, one retrospective analysis of 355 lung cancer cases (42) found young women to be diagnosed at a significantly later stage than other patient populations. Consequently, young individuals often have limited local therapeutic options such as curative-intent surgeries available to them (43). Indeed, a U.S. SEER database review from 2010-2017 (44) found that patients under 50 years old comprised only 6.6% of 33,586 surgically treated patients with NSCLC, similar to how patients under 50 encompassed only 5.0% of 11,663 surgical cases in a 2004 Japanese Lung Cancer Registry Study (44). Further, a nationwide South Korean database study found a significant decrease in the frequency of surgery among individuals aged 20–60 years from 2015 (45). Such data is unfortunate, considering how young patients exhibit significantly enhanced surgical outcomes compared to their older counterparts (44, 46).

**Figure 1:**
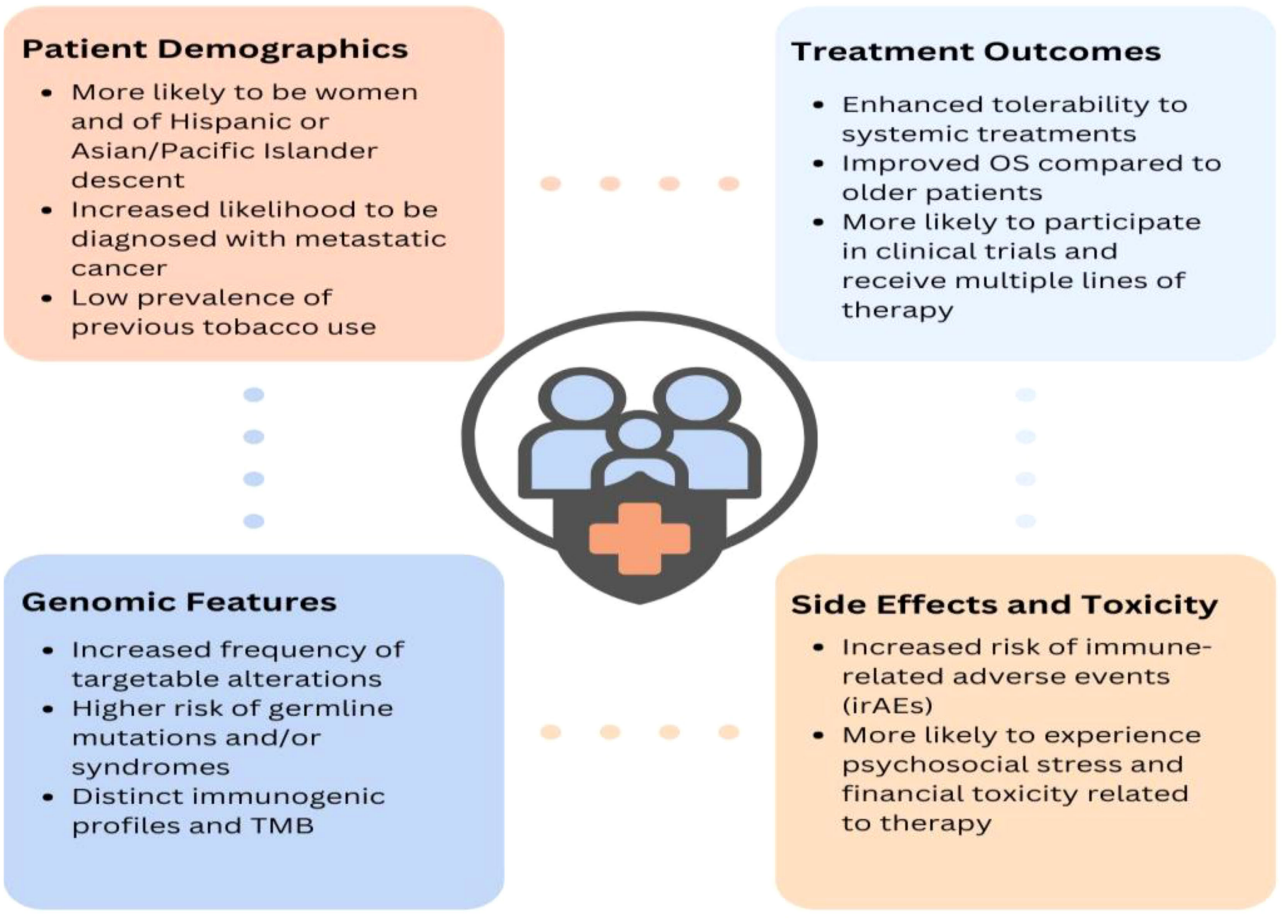
Clinical characteristics of young lung cancer.

In addition to later-stage diagnoses, there remain numerous other unique clinical characteristics of young lung cancer. A study of 5,657 patients with lung cancer (41) within the National Cancer Data Base found young patients (ages 20-46) to have a significantly higher prevalence of adenocarcinoma than older patients (49% vs. 39%). Young patients with lung cancer are also significantly more likely to be female (4, 47) or Asian/Pacific Islander (48), have no tobacco use history (49), metastasis to the brain (50), and a higher frequency of somatic mutations or rearrangements such as EGFR, ALK, RET, and ROS1 (49, 51, 52) (**Figure 1**). Indeed, an evaluation of 2,237 patients with a diagnosis of non-small cell lung cancer (NSCLC) from 2002 to 2014 at Dana-Farber Cancer Institute (DFCI) found that tumors from patients <50 at diagnosis were 59% more likely to harbor a targetable alteration (52). Notably, young patients, especially women, demonstrate improved survival (37), particularly in earlier stages (41). This may be attributed to their relative lack of comorbidities, higher frequency of targetable mutations, and capacity to tolerate more extensive surgical interventions and lines of therapy than their older counterparts.

A recent systemic review (53) evaluated diagnostic time intervals and their effect on prognosis of patients with lung cancer. These intervals included time from symptom onset to treatment, symptom onset to diagnosis, first specialist visit to diagnosis, specialist referral to surgery or treatment, and time from diagnosis to treatment. Interestingly, 35% of time intervals studied showed no relationship between waiting time and the disease prognosis, while 37.5% of time intervals studied found a better prognosis with longer time intervals, and 27.5% found a better prognosis with shorter time intervals. To explain these paradoxical results (which have been replicated) (54), it has been suggested that earlier-stage diagnoses may require more testing or evaluation, thereby prolonging the diagnostic interval, but correlating with improved prognosis due to earlier disease stage and more targeted or curative-intent treatment options. While diagnostic time intervals may be an inconsistent measure of prognosis and survival, these studies denote the importance of early detection and intervention, given that earlier stage at diagnosis was a favorable prognostic modifier across studies. Therefore, early detection of lung cancer remains of paramount importance, particularly among young patients who tend to be diagnosed with later-stage disease.

Studies such as the Taiwan Lung Cancer Screening in Never-Smoker Trial (TALENT) (55) and the FANSS study (56) have explored the efficacy of low-dose CT scans among individuals with no tobacco use. Favorable preliminary results reveal diagnoses of early-stage lung cancer with adequate management. Most participants diagnosed with lung cancer in the TALENT study (246/318) had stage I disease. Similarly as promising, in the FANSS study, eight patients with a screening CT scan showing Lung-RADS 3 and 4 (57) lesions remain in close follow-up; three who were diagnosed with invasive adenocarcinoma all underwent surgical resection and are receiving adjuvant targeted therapy. Notably, TALENT also found that individuals with a family history of lung cancer were *significantly* more likely to develop lung cancer than those without a family history (3.2% vs 2.0%; p<0.001). This risk was positively correlated with the number of first-degree relatives with lung cancer, particularly a mother or sibling. Such remarkable feasibility of early lung cancer detection among non-tobacco users, compounded with newfound knowledge of family history, offers an attractive opportunity for deployment of lung cancer screening among young patients (especially those with a family history of lung cancer), given their historically advanced presentation of disease and likelihood of being excluded from current screening guidelines based on age and lack of tobacco use history. The EQUAL study is also deploying a novel ctDNA assay to detect early-stage EGFRm-NSCLC among non-tobacco using East/Southeast Asian and Hispanic/Latinx individuals who would otherwise be ineligible for lung cancer screening despite being at higher risk for the disease due to their race or ethnicity. Among a sample size of 1,000 40-80-year-olds, the study seeks to recruit 500 40-49-year-olds, most of whom will have a first-degree family history of EGFRm-NSCLC. This recruitment strategy will facilitate a preliminary risk stratification based on family history of EGFR lung cancer (58).

Still, to modify or develop new guidelines, the United States Preventative Services Task Force (USPSTF) first will review nominated topics based on their relevance to prevention and primary care, importance for public health, potential impact, and novel evidence. A draft research plan is then formulated, posted, and commented on, whereafter the USPSTF will refine and finalize the research plan that will guide the development of a draft recommendation statement. Following a 4-week public comment period, the USPSTF will refine, finalize, and post the updated recommendations (59).

## 3 Genomics of young lung cancer

As previously mentioned, younger age at diagnosis of lung cancer is strongly associated with presence of targetable alterations, making early biomarker testing critical. One global prospective review (49) of patients <40 years old found that of 112 individuals, 84% of those with adenocarcinoma had a targetable alteration, including 85% of those with stage IV disease Descriptive studies of oncogenic genomic alterations in this population have similarly found 52-91% of patients to have targetable alterations (51, 52, 60–69).

In one of the largest retrospective studies analyzing data from over 8,000 patients with NSCLC, the most common single nucleotide variants (SNV) among 189 patients under 50 years old were TP53 (57.1%), KRAS (15.5%), EGFR (21.4%), STK11 (10.1%), SMARCA4 (7.7%), and LRP1B (4.8%) (70, 71). Though specific types of KRAS mutations were not specified, transition mutations such as KRAS G12D mutations are more common in individuals without tobacco use (72) and may therefore be more enriched among young patients. In that same study, the most common fusions or skipping variants among patients 151 patients under 50 years old were ALK (14.6%), RPS6KB1 (3.3%), ROS1 (3.3%), RET (2%), and MET (2%). Indeed, over 20% of young patients have been found to have at least one alterations in ALK (52, 62, 68, 73–75), ROS, RET, or NTRK (76), a frequency nearly four times higher than in older patients (77). Among these, ALK rearrangements are the most commonly identified, with studies globally reporting variable prevalence with rates between 10-41% in young patients and higher rates among young Asians (12, 52, 62, 64, 65, 69). The EML4-ALK fusion, specifically, has been found to be more prevalent in younger patients with lung cancer, though this was a much rarer genetic alteration among all patients studied (70). Other genetic alterations more common among younger patients include RAD51B, CREBBP, LZTR1 SNVs as well as ROS1 and NTRK1 fusions (70). Conversely, KRAS SNVs, particularly KRAS G12C, MET exon 14 skipping variant (70), and BRAF V600E mutations (52), are distinctly more common among older patients.

Studies report conflicting results on whether EGFR mutation frequency in young patients differs compared to older populations. Among 14 studies analyzing EGFR mutation frequency, 7 found no differences between these populations, 3 reported higher rates among younger populations, and 4 reported higher rates among older populations (69). Regardless, rates of EGFR mutations among younger populations remain high, ranging from 12.8%-60%, with higher rates reported in studies conducted among Asians and Hispanic patients (69). Some studies suggest that the type of EGFR mutation may vary between older and younger populations (78), with EGFR exon 19 deletions (49, 70) and exon 20 insertions being more common among young patients, and EGFR L858R and *de novo* T790M more common among older populations (62, 69, 73, 75, 79, 80). Wu et al. (79, 80) has also described that uncommon EGFR mutations (defined as EGFR mutations other than the tyrosine kinase inhibitor (TKI)-sensitizing EGFR exon 19 deletion or L858R mutations) are more frequent among young patients (18% vs 9%; p = 0.02; 13.7% vs 8.4%; p = 0.03, respectively), suggesting that higher rates of such uncommon EGFR non-canonical mutations in Asian patients diagnosed under 50 years old may lead to lower response rates in these younger populations, at least partly due to limited treatment options compared to individuals with classical TKI-sensitizing EGFR mutation types. Along those lines, Hou et al. noted younger Chinese patients with EGFR mutations’ increased likelihood of harboring concurrent TP53 mutations, a negative prognostic factor for treatment response to EGFR TKIs (13, 81). Young patients with lung cancer may also be more likely to have underlying germline or hereditary TP53 mutations from syndromes such as Li-Fraumeni Syndrome (82), though it is unclear if germline TP53 mutation confers an increased risk of treatment resistance in this context (13).

To further understand disease characteristics and germline contribution among this population, two studies are ongoing at DFCI, one specifically investigating NSCLC or SCLC in those 45 and under (Biology of Young Lung Cancer, NCT05265429), and one studying lung cancers with strong family histories (the INHERIT study) (83), both of which incorporate clinical and exploratory research germline testing in addition to exposure and somatic data. Study results will attempt to aid in improving screening, early detection, prevention, and treatment of patients, as little is currently known regarding the risk factors, survival rates, and clinical, genomic, and histopathological characteristics of young-onset lung cancer. Similarly, the ongoing 23andMe Lung Cancer Genetics Study (84) aims to recruit 10,000 patients with lung cancer across the U.S. to provide further insights genetics of people diagnosed with lung cancer.

Younger patients with lung cancer also have distinct immune profiles and tumor mutational burden (TMB), related to the high prevalence of targetable driver alterations (e.g., EGFR mutations and oncogene fusions) (70). Although tumors that harbor targetable driver alterations are often PD-L1 positive, PD-L1 positivity in these tumors is believed to result from intrinsic oncogene-driven activation of downstream signaling effectors, such as STAT3 and HIF1α, which transcriptionally upregulate PD-L1 expression (85, 86). This mechanism differs from true T cell-mediated immunogenicity via IFNγ signaling, which is typically associated with responsiveness to immunotherapy. One study of 97 patients with NSCLC who were under 65 years old in China found these patients to have a lower expression of immune-related genes indicating reduced immune system activation, suggesting a lower response to immunotherapy (immune checkpoint inhibitors/ICIs), though PD-L1 expression level among younger and older patients with NSCLC was similar (70) (this study did not control for tobacco exposure). This trend was more pronounced among younger males, who had worse survival when receiving pembrolizumab alone in comparison to pembrolizumab with chemotherapy. Although there were no significant differences in the OS and progression free survival (PFS) between younger and older patients and between males and females, younger females had improved outcomes and better PFS when immunotherapy was combined with chemotherapy instead of immunotherapy monotherapy (70). Nevertheless, compared to older females, younger females had a similar outcome when treated with immunotherapy alone (70).

While the aforementioned study also found that both TMB and TIGS scores (signature for tumor inflammation) decreased significantly with decreasing age (p < 2E-4) and were significantly lower in almost all age groups compared against the ≥65 group (70), among patients with high TMB, another study of 93 patients under 65 years old found among them a higher prevalence of KRAS and STK11 co-mutations (71). Mutations in STK11 and KEAP1 generally correlate with worse outcomes among patients with concurrent mutations in KRAS who received ICIs (87, 88), further highlighting the importance of biomarker testing and considering the potential impact of co-mutations in young patients with lung cancer. Patients with concurrent mutations in KEAP1, STK11, SMARCA4 and PBRM1 have also been found to have a low response to immunotherapy, despite having a high TMB (89).More research is warranted to investigate the prognosis of patients with these mutations and to further understand frequent co-mutation patterns in young patients. To aid in this, researchers at DFCI are developing a longitudinal, international cohort study to track cases of young lung cancer globally and use data from the evolving cohort to provide insights into the molecular background, risk factors, optimal treatment, and follow-up care for young patients with lung cancer. The aforementioned Biology of Young Lung Cancer study also seeks to collect EHR data, blood, and tissue samples in patients 45 years old and younger to better estimate lung cancer risks and potential risk factors for the disease, as well as the somatic and germline genetic changes that may be shared among young patients (90). Researchers aim to routinely disseminate interim results of these cohorts through scientific meeting presentations and peer-reviewed publications so that data may serve as an impetus for the implementation of tailored diagnostic, treatment, and management services for young patients with lung cancer across the globe. Numerous other studies are also ongoing to investigate potential risk factors, needs, and clinical outcomes among young patients with lung cancer (**Table 1**).

**Table 1:** Ongoing studies to investigate risk factors, needs, and outcomes among young patients with lung cancer.

NCI Number	Title	Purpose	Components	Start
NCT04640259	EoYLC: ALCMI Epidemiology of Young Lung Cancer Study	Explores environmental exposures, childhood exposures, and other risk factors for lung cancer in individuals <50 years old.	- Survey - Blood draw	2021
NCT05587439	INHERIT: Investigating Hereditary Risk In Thoracic Cancers study	Seeks to discover genetic contributors to lung cancer development and establish a repository for clinicopathologic information and biologic specimens from individuals with inherited lung cancer predisposition.	- Questionnaires - Medical record review - Blood sample - Saliva sample - Optional release of tissue samples	2023
NCT05265429	Biology of Young Lung Cancer Study: The YOUNG LUNG Study	Seeks to understand causes of and risk factors for lung cancer in individuals 45 years old and younger, examine somatic or germline) genetic changes that may be shared among young patients with lung cancer, and improve opportunities for screening and treatment in this population.	- Questionnaires - Medical record review - Blood and/or saliva samples - Optional tumor tissue samples	2023
--	Young Lung: Psychosocial Needs Assessment	Explores the financial toxicity and emotional, physical, social, and functional well-being of patients with lung cancer who are 50 years and younger, in addition to comparing these needs to those of older patients. This study’s results formed the basis for the Young Lung Cancer Clinical and Research Program at Dana-Farber Cancer Institute, the first program dedicated to this patient population in the nation.	- Questionnaire - Optional focus groups	2022
--	Y-Lung Global Registry	Seeks to investigate environmental, genetic, and occupational factors to elucidate the mechanisms of tumor carcinogenesis in young patients with lung cancer, particularly those under 45 years old, who often have never smoked and are not exposed to known environmental carcinogens.	-	
--	Global eNRGy1 Registry	Aims to characterize NRG1 fusion-positive lung cancers in the largest and most diverse series to date.	- Profiling data: DNA-based and/or RNA-based NGS and FISH. - Collection of anonymized clinical, pathologic, molecular, and response data	2018
NCT06532149	EROS: ERectile Dysfunctions, gOnadotoxicity and Sexual Health Assessment in Men With Lung Cancer	Explores the incidence of endocrine toxicity and sexual dysfunction in male patients aged 18-75 receiving active treatment for NSCLC.	- Questionnaire - Blood sample	2024
NCT02273336	Comprehensive Genomic Analysis in Tissue and Blood Samples From Young Patients With Lung Cancer	Seeks to perform a comprehensive genomic analysis of young patients with lung cancer’s samples to facilitate delivery of targeted therapies and clinical trial enrollment, to characterize the impact of young age at lung cancer diagnosis on the genomic landscape of primary lung cancer, and to establish a prospective registry of young lung cancer patients for both tumor and germline next generation sequencing.	- Blood sample - Tissue sample	2014
NCT04551378	The Effect of COVID-19 Pandemic on Adolescent and Young Adult Cancer Patients and Survivors	Investigates how the COVID-19 pandemic has impacted the psychological, financial, physical, and social well-being of adolescent and young adult patients diagnosed with cancer between the ages of 15-39.	- Questionnaires	2020
NCT06904365	Ovarian-Sparing Adaptive Radiotherapy in Young Adult Women (OvAR-Y): an In-Silico Feasibility Trial	Investigates the ovarian visualization incidence of dosimetrically sparing one or both ovaries from a functionally ablative radiation dose following CBCT sessions in patients 18-50 years old receiving radiation therapy for any indication .	- Radiation therapy - Pelvic imaging	2025
NCT05228275	Evaluation of Immunologic Response Following COVID-19 Vaccination in Children, Adolescents, and Young Adults With Cancer	Aims to characterize the immunologic response following the COVID-19 vaccine in children, adolescents, and young adults with cancer who are currently receiving or who recently completed treatment with immunosuppressive therapy and are 37 years old or younger	- Surveys - COVID-19 vaccination(s) - Blood samples	2022

## 4 Treatment of young lung cancer

The unique epidemiologic, biologic, and diagnostic considerations in younger-onset lung cancer highlight the need for tailored treatment approaches (50). Young patients are more likely to be diagnosed with brain metastases (50) and tend to harbor larger tumors, more nodal involvement, and initial diagnosis of metastatic disease (13, 41). However, their significantly higher rates of actionable alterations (49) highlight the crucial need for early genomic testing to guide therapy selection (91–97). Given the significant enrichment for fusion-positive lung cancers in younger populations, RNA-based sequencing is necessary to elucidate targets when DNA-based testing is uninformative, as the genomic breakpoints for novel ALK fusions and more rare fusions (e.g, those in ROS1, TRK, and NRG1) often occur within intronic sequences that are long or contain repetitive elements and are not adequately covered in targeted DNA-based panels. To account for this, RNA-based NGS using anchored multiplex PCR (e.g., Archer FusionPlex) has been used to identify both known and novel fusion partners for ALK, ROS1, RET, NRG1, and TRK. In a study out of MSK-IMPACT using Archer-based technology, a custom RNA-sequencing panel was able to detect fusions as well as MET exon 14 alterations in 14% of cases negative for oncogenic drivers by DNA-based NGS (PMID 31028088). RNA-based sequencing also detects oncogenic fusions in cases where tumor purity may be too low for DNA detection, owing to the high level of expression of fusion proteins.

As previously mentioned, analyses continue to demonstrate the improved OS of young patients with lung cancer (41, 50, 67, 98–100). This is likely a result of their higher prevalence of targeted alterations, increased likelihood of receiving aggressive treatments (41), multimodal therapy (41, 98, 101), and surgical resection in early stage and oligometastatic disease (102–107), as well as their receiving of additional lines of treatment given their enhanced performance status (41, 50, 108) and greater likelihood of participating in clinical trials (109). While surgery has historically not been an option for patients with metastatic lung cancer, there has been a shift in those with oligometastatic disease. More recent studies, with a median age of 61, have shown improved PFS and OS in oligometastatic patients (102–104), with significantly improved outcomes (HR 0.4) among patients under 60 years old (105). Older, retrospective analyses suggesting worse outcomes in young patients with targetable alterations (110) are likely due to these studies pre-dating newer generation and highly effective TKIs such as osimertinib, lorlatinib, and repotrectinib [**Table 2** (111)].

**Table 2:** Common NSCLC treatment for young patients.

Treatment	Disease Profile	Type	Purpose	Dose/Administration	Toxicity Profile
Carboplatin-paclitaxel (149)	NSCLC, stages III-IV	Chemotherapy	• 1L treatment in combination with carboplatin, for patients who are not candidates for curative surgery or radiation	• Carboplatin intravenous infusion over 60 minutes, paclitaxel intravenous infusion over 3 hours	• Leukopenia, anemia, Hair loss, constipation, decreased appetite
Pemetrexed	NSCLC, stages IIIB-IV	Chemotherapy	• 1L treatment + cisplatin for patients with locally advanced or metastatic non-squamous NSCLC • As a single agent for the maintenance treatment of patients with locally advanced or metastatic, non-squamous NSCLC whose disease has not progressed after 4 cycles of platinum-based 1L chemotherapy. • As a single agent for the treatment of patients with recurrent, metastatic non-squamous, NSCLC after prior chemotherapy	• Administered as a single agent or with cisplatin, in patients with creatinine clearance of ≥45 mL/minute, as a 500 mg/m2 intravenous infusion over 10 minutes on D1 of each 21-day cycle.	• When administered as a single agent: fatigue, nausea, anorexia • When administered with cisplatin: vomiting, neutropenia, anemia, stomatitis/pharyngitis, thrombocytopenia, constipation
Adagrasib	NSCLC, stages IIIB-IV	TKI	• Treatment for patients with KRAS G12C NSCLC who have received **≥** prior systemic therapy	• 600 mg orally, twice daily	• Anemia, neutropenia, thrombocytopenia, alopecia, peripheral neuropathy, nausea, fatigue
Alectinib	NSCLC, stages IB-IIIA	TKI	• Adjuvant treatment for patients with ALK-positive NSCLC	• 600 mg orally, twice daily	• Hepatotoxicity, constipation, fatigue, myalgia, edema, rash, cough
Lorlatinib	NSCLC, stage IV	TKI	• Treatment for patients whose metastatic disease has progressed on another 1L TKI for ALK-positive NSCLC	• 100 mg orally, once daily	• Edema, peripheral neuropathy, cognitive effects, dyspnea, fatigue, weight gain, arthralgia, mood effects, diarrhea
Osimertinib	NSCLC, stages IIB-IV	TKI	• Adjuvant treatment for patients with EGFRm-NSCLC (exon 19 deletions or exon 21 L858R mutations) • 1L treatment for patients with EGFRm-NSCLC (exon 19 deletions or exon 21 L858R mutations) • Treatment for patients with metastatic EGFRm-NSCLC (T790M mutation), whose disease has progressed on or after another EGFR TKI	• Adjuvant: 80mg orally, once daily, for ≤3 years • Metastatic: 80mg orally, once daily	• Leukopenia, lymphopenia, thrombocytopenia, diarrhea, anemia, rash, musculoskeletal pain, nail toxicity, neutropenia, dry skin, stomatitis, fatigue, cough
Repotrectinib	NSCLC, stages IIIB-IV	TKI	• Treatment for patients with locally advanced or metastatic ROS1 or NTRK-positive NSCLC	• 160 mg orally, once daily for 14 days, then increase to 160 mg, twice daily	• Dizziness, dysgeusia, peripheral neuropathy, constipation, dyspnea, fatigue, ataxia, cognitive impairment, muscular weakness, nausea
Erlotinib	NSCLC, stage IV	TKI	• Treatment for patients with metastatic EGFRm-NSCLC (exon 19 deletions or exon 21 L858R substitution mutations) after progression following **≥1** prior chemotherapy regimen	• 150mg orally, once daily	• Rash, diarrhea, anorexia, fatigue, dyspnea, cough, nausea, vomiting
Immunotherapy	NSCLC, stages III-IV	Immunotherapy	• Adjuvant or metastatic treatment for patients with NSCLC or advanced SCLC	Intravenous infusion	• Cough, fatigue, pneumonitis, upper respiratory tract infections, dyspnea, rash, decreased appetite

For those with EGFR-mutant disease, osimertinib, a third generation EGFR TKI, significantly improved OS and progression-free survival (PFS) in newly diagnosed patients, with a median OS of 38.6 months (91) and a median PFS of 18.9 months (112). More recently, osimertinib has been assessed in combination with chemotherapy (platinum agent plus pemetrexed) in the front-line setting. Compared to osimertinib alone, the combination significantly improved PFS [HR for disease progression or death, 0.62; median response duration of 24.0 months vs single agent osimertinib 15.3 months (113)]. A consistent benefit was observed among young and older patients, though individuals under age 65 appeared to derive potentially greater benefit with the combination than with osimertinib alone (HR 0.59 (0.44-0.80) vs 0.68 (0.47-0.98 in those over 65). However, OS data is still immature and requires longer follow up to determine the survival benefit of adding chemotherapy to osimertinib. Other intensification regimens over first-line osimertinib monotherapy have also been developed, including the combination of amivantamab plus lazertininb in the MARIPOSA trial (114), as well as the addition of VEGF inhibitors to osimertinib in the RAMOSE trial (115), both of which significantly prolonged PFS compared to osimertinib alone. Further research to understand the benefits of these intensification regimens is vital, as young patients with lung cancer are more likely to receive and tolerate these combination therapies. To aid in this, numerous clinical trials are ongoing to investigate novel drugs and therapeutic combinations for genomically driven lung cancer (**Table 3**).

**Table 3:** Ongoing phase II and III clinical trials for genomically-driven lung cancer.

Trials for EGFR Mutations			
NCI Number	Title	Genomic Alteration	Stage
NCT03122717	A Phase 1/2 Study of Osimertinib in Combination With Gefitinib in EGFR Inhibitor naïve Advanced EGFR Mutant Lung Cancer	Ex19del, L858R	IV
NCT03392246	A Phase 2 Study of Osimertinib in Combination With Selumetinib in EGFR Inhibitor naïve Advanced EGFR Mutant Lung Cancer	Ex19del, L858R	IV
NCT03974022	A Phase I/II, Open-Label, Multicenter Study to Assess the Safety, Tolerability, Pharmacokinetics and Anti-tumor Efficacy of DZD9008 in Patients with Advanced NSCLC with EGFR or HER2 Mutation	Ex20ins, HER2	IIIB/IV
NCT04965090	A Phase 2 Single-Arm Study of Amivantamab (JNJ-61186372) and Lazertinib in Metastatic EGFR-mutant Lung Cancer With Progressive or New CNS Metastases on Previous Treatment	Somatic sensitizing mutation	IV, recurrent
NCT03944772	A Biomarker-directed Phase 2 Platform Study in Patients With Advanced Non-Small Lung Cancer Whose Disease Has Progressed on First-Line Osimertinib Therapy	Any sensitizing mutation	IIIB-IV
NCT05526755	An Open-label, Single-arm, Phase II, Multinational, Multicentre Study to Assess the Efficacy and Safety of 5 Years of Osimertinib in Participants With EGFRm-positive Stage II-IIIB NSCLC, Following Complete Tumour Resection With or Without Adjuvant Chemotherapy	Ex19del, L858R, either alone or in combination with another EGFR mutation	II-IIIB
NCT03433469	A Phase II Study to Evaluate Neoadjuvant Osimertinib Therapy in Patients With Surgically Resectable, EGFR-Mutant Non-Small Cell Lung Cancer	Ex19del, L858R, T790M	I-IIIA
NCT02511106	A Phase III, Double-blind, Randomized, Placebo-controlled Multi-centre, Study to Assess the Efficacy and Safety of AZD9291 Versus Placebo, in Patients With Epidermal Growth Factor Receptor Mutation Positive Stage IB-IIIA Non-small Cell Lung Carcinoma, Following Complete Tumour Resection With or Without Adjuvant Chemotherapy (ADAURA)	Ex19del, L858R, either alone or in combination with another EGFR mutation	IB-IIIA
NCT04035486	A Phase III, Open-label, Randomized Study of Osimertinib With or Without Platinum Plus Pemetrexed Chemo, as First-line Treatment in Patients With Epidermal Growth Factor Receptor (EGFR) Mutation Positive, Locally Advanced or Metastatic Non-small Cell Lung Cancer (FLAURA2)	Ex19del, L858R, either alone or in combination with another EGFR mutation	IIIB-IV
NCT06396065	A Randomized, Double-blind, Multi-center, Phase III Study of AK112 or Placebo Combined with Pemetrexed and Carboplatin in Patients with EGFR-mutant Locally Advanced or Metastatic Non-squamous NSCLC Who Have Failed to EGFR-TKI Treatment	Any activating mutation	IIIB-IV
NCT02438722	A Randomized Phase II/III Trial of Afatinib Plus Cetuximab Versus Afatinib Alone in Treatment-Naive Patients With Advanced, EGFR Mutation Positive NSCLC	Ex19del, L858R, T790M, alone or in combination with another EGFR mutation	IV, recurrent
NCT03667820	Phase II Trial of Osimertinib in Combination With Stereotactic Ablative Radiation (SABR) in EGFR Mutant Advanced NSCLC	Ex19del, Ex21del	IIIB-IV
NCT04606771	A Multi-centre Phase II, Double-Blind, Randomised Study of Savolitinib in Combination With Osimertinib vs Savolitinib in Combination With Placebo in Patients With EGFRm+ and MET Amplified Locally Advanced or Metastatic Non-Small Cell Lung Cancer Who Have Progressed Following Treatment With Osimertinib	Any sensitizing mutation	IIIB-IV
NCT03940703	A Phase II, Two-arm Study to Investigate Tepotinib Combined With Osimertinib in MET Amplified, Advanced or Metastatic NSCLC Harboring Activating EGFR Mutations and Having Acquired Resistance to Prior Osimertinib Therapy (INSIGHT 2)	Any activating mutation	IIIB-IV
NCT03831932	A Phase Ib Study of Osimertinib (AZD9291) and Telaglenastat (CB-839) HCl in Patients With EGFR Mutant Non-Small Cell Lung Cancer	L858R, Ex19del, alone or in combination with another EGFR mutation	IV
NCT04538664	A Randomized, Open-label Phase 3 Study of Combination Amivantamab and Carboplatin-Pemetrexed Therapy, Compared With Carboplatin-Pemetrexed, in Patients With EGFR Exon 20ins Mutated Locally Advanced or Metastatic Non-Small Cell Lung Cancer	Ex20ins	IIIB-IV
NCT04487080	A Phase 3, Randomized Study of Amivantamab and Lazertinib Combination Therapy Versus Osimertinib Versus Lazertinib as First-Line Treatment in Patients With EGFR-Mutated Locally Advanced or Metastatic Non-Small Cell Lung Cancer	Ex19del, L858R	IIIB-IV
NCT04351555	A Phase III, Randomised, Controlled, Multi-center, 3-Arm Study of Neoadjuvant Osimertinib as Monotherapy or in Combination With Chemotherapy Versus Standard of Care Chemotherapy Alone for the Treatment of Patients With Epidermal Growth Factor Receptor Mutation Positive, Resectable Non-small Cell Lung Cancer	L858R, Ex19del, alone or in combination with another EGFR mutation	II-IIIB N2
NCT03381274	A Multiarm, Open-label, Multicenter, Phase 1b/2 Study to Evaluate Novel Combination Therapies in Subjects With Previously Treated Advanced EGFRm NSCLC	Any mutation	IIIB-IV
NCT01553942	Afatinib Sequenced With Concurrent Chemotherapy and Radiation in EGFR-Mutant Non-Small Cell Lung Tumors: The ASCENT Trial	Any mutation	IIIA
NCT04545710	A Phase II Trial of Osimertinib and Abemaciclib With a Focus on Non-Small Cell Lung Cancer Patients With EGFR Activating Mutations With Osimertinib Resistance	Exon 21 L858R, Exon 19 deletion, Exon 18 G719X, Exon 21 L861Q-ACTIVATING	IV, recurrent
NCT04619004	HERTHENA-Lung01: A Phase 2 Randomized Open-Label Study of Patritumab Deruxtecan (U3-1402) in Subjects With Previously Treated Metastatic or Locally Advanced EGFR-mutated Non-Small Cell Lung Cancer	Ex19del, L858R	IIIB-IV
NCT02411448	A Multicenter, Randomized, Double-Blind Study of Erlotinib in Combination With Ramucirumab or Placebo in Previously Untreated Patients With EGFR Mutation-Positive Metastatic Non-Small Cell Lung Cancer	Ex19del, L858R	IV
NCT04988295	A Phase 3, Open-Label, Randomized Study of Amivantamab and Lazertinib in Combination With Platinum-Based Chemotherapy Compared With Platinum-Based Chemotherapy in Patients With EGFR-Mutated Locally Advanced or Metastatic Non-Small Cell Lung Cancer After Osimertinib Failure	Ex19del, L858R	IIIB-IV
NCT03292133	A Phase 2 Study of EGF816 and Gefitinib in TKI-naïve EGFR-mutant Non-Small Cell Lung Cancer	L858R, Ex19del (with exceptions)	IIIB, IV, recurrent
NCT03066206	A Phase II Study of Poziotinib in EGFR or HER2 Mutant Advanced Solid Tumors	Exon 20 in-frame insertion or point mutation excluding T790M	IV, recurrent
NCT03833154	A Phase III, Randomized, Placebo-controlled, Double-blind, Multi-center, International Study of Durvalumab With Stereotactic Body Radiation Therapy (SBRT) for the Treatment of Patients With Unresected Stage I/II, Lymph-node Negative Non-small Cell Lung Cancer (PACIFIC-4/RTOG-3515) Osimertinib Following SBRT, a Single Arm Cohort for Patients With Unresected Stage I/II, Lymph Node Negative NSCLC Harboring a Sensitizing EGFR Mutation	Ex19del, L858R	Stage I-II lymph node-negative
NCT02193282	Randomized Study of Erlotinib vs Observation in Patients With Completely Resected EGFR Mutant NSCLC	Ex19del, L858R	IB-IIIA
NCT03944772	A Biomarker-directed Phase 2 Platform Study in Patients With Advanced Non-Small Lung Cancer Whose Disease Has Progressed on First-Line Osimertinib Therapy.	Any sensitizing mutation	IIIB-IV
NCT04765059	A Phase III, Randomized, Double-Blind, Placebo-Controlled Study of Platinum Plus Pemetrexed Chemotherapy Plus Osimertinib Versus Platinum Plus Pemetrexed Chemotherapy Plus Placebo in Patients With EGFRm, Locally Advanced or Metastatic NSCLC Who Have Progressed Extracranially Following First-Line Osimertinib Therapy (COMPEL)	Ex19del, L858R, alone or in combination with another EGFR mutation	IIIB-IV, recurrent
NCT03521154	A Phase III, Randomized, Double-blind, Placebo-controlled, Multicenter, International Study of Osimertinib as Maintenance Therapy in Patients With Locally Advanced, Unresectable EGFR Mutation-positive Non-Small Cell Lung Cancer (Stage III) Whose Disease Has Not Progressed Following Definitive Platinum-based Chemoradiation Therapy (LAURA).	Ex19del, L858R, alone or in combination with other EGFR mutations	III
NCT05607550	A Global, Phase 3, Randomized, Multicenter, Open-Label Study to Investigate the Efficacy and Safety of Furmonertinib Compared to Platinum-Based Chemotherapy as First-Line Treatment for Patients With Locally Advanced or Metastatic Non-Small Cell Lung Cancer (NSCLC) With EGFR) Exon 20 Insertion Mutations (FURVENT)	Ex20ins	IIIB-IV
NCT04036682	A Phase 1/2, Open-Label, Multi-Center Trial to Assess Safety, Tolerability, Pharmacokinetics, Pharmacodynamics, and Efficacy of CLN-081 in Patients With Locally-Advanced or Metastatic Non-Small Cell Lung Cancer Harboring EGFR Exon 20 Insertion Mutations Who Have Previously Received Platinum-Based Systemic Chemotherapy	Ex20ins	IIIB-IV
NCT04129502	A Randomized Phase 3 Multicenter Open-Label Study to Compare the Efficacy of TAK-788 as First-Line Treatment Versus Platinum-Based Chemotherapy in Patients With Non-Small Cell Lung Cancer With EGFR Exon 20 Insertion Mutations	Ex20ins	IIIB-IV, recurrent
NCT05099172	An Open Label, First-in-human Study of BAY 2927088 in Participants With Advanced NSCLC Harboring an EGFR and/or HER2 Mutation	Activating EGFR mutation	IIIB-IV, recurrent
NCT02971501	A Phase II Trial of Osimertinib (AZD9291) With or Without Bevacizumab in Patients With EGFR Mutation Positive NSCLC and Brain Metastases	Activating EGFR mutation	IV
NCT05388669	A Phase 3, Open-label, Randomized Study of Lazertinib With Subcutaneous Amivantamab Compared With Intravenous Amivantamab in Patients With EGFR-mutated Advanced or Metastatic Non-small Cell Lung Cancer After Progression on Osimertinib and Chemotherapy	Ex19del, L858R	IV
NCT05017025	A Phase Ib/II Trial to Evaluate Safety, Tolerability and Efficacy of Aurora Kinase Inhibitor LY3295668 in Combination With Osimertinib for Patients With EGFR-Mutant Non-Small Cell Lung Cancer	Ex19del, L858R	IIIB-IV
NCT03410043	Randomized Phase II Trial of Osimertinib With or Without Local Consolidation Therapy (LCT) for Patients With EGFR-Mutant Metastatic NSCLC (NORTHSTAR)	L858R, Ex19del, acquired T790M	IIIB-IV, recurrent
NCT05338970	A Phase 3, Randomized, Open-label Study of Patritumab Deruxtecan Versus Platinum-based Chemotherapy in Metastatic or Locally Advanced Epidermal Growth Factor Receptor-mutated (EGFRm) Non-small Cell Lung Cancer (NSCLC) After Failure of EGFR TKI Therapy (HERTHENA-Lung02)	Ex19del, L858R	IIIB-IV,
NCT03909334	An Open-Label Randomized Phase II Study of Combining Osimertinib With and Without Ramucirumab in TKI-naïve EGFR-Mutant Locally Advanced or Metastatic NSCLC	Ex19del, L858R	IIIB-IV
NCT04285671	UCLA L-08: A Phase Ib/II Study of Combined HER Inhibition Adding Necitumumab and Trastuzumab to Osimertinib in Patients With Refractory EGFR-Mutated Lung Cancer	activating and sensitizing EGFR mutation	IV
NCT04120454	An Investigator-Sponsored Phase 2 Single Arm Trial of Ramucirumab and Pembrolizumab in Patients With EGFR Mutant Non-Small Cell Lung Cancer	Sensitizing EGFR mutations (excl. ex20 mutations)	IV, recurrent
NCT02917993	An Open-Label Phase 1/2 Study of Itacitinib in Combination With Osimertinib in Subjects With Locally Advanced or Metastatic Non-Small Cell Lung Cancer	somatic activating mutation in EGFR	IIIB-IV
Trials for ALK, NTRK, or RET alterations			
Trial Number	Title	Genomic Alteration	Stage
NCT03202940	A Phase IB/II Study of Alectinib Combined With Cobimetinib in Advanced ALK+ NSCLC	ALK rearrangement	IV
NCT02767804	Phase 3 Randomized Study Comparing X-396 (Ensartinib) to Crizotinib in ALK Positive NSCLC Patients	ALK	IIIB-IV, recurrent
NCT03052608	A Phase 3, Randomized, Open-Label Study of Lorlatinib (PF-06463922) Monotherapy versus Crizotinib Monotherapy in the First-Line Treatment of Patients with Advanced ALK-positive NSCLC	ALK	IIIB-IV
NCT03194893	A Multicenter, International, Rollover Study of Alectinib in Patients With Anaplastic Lymphoma Kinase (ALK)-Positive or RET-Positive Cancer	ALK, RET	Variable
NCT02568267	An Open-Label, Multicenter, Global Phase 2 Basket Study of Entrectinib for the Treatment of Patients With Locally Advanced or Metastatic Solid Tumors That Harbor NTRK1/2/3, ROS1, or ALK Gene Rearrangements	NTRK1/2/3, ROS1, or ALK	IIIB-IV
NCT03456076	A Phase III, Open-Label, Randomized Study to Evaluate the Efficacy and Safety of Adjuvant Alectinib Versus Adjuvant Platinum-Based Chemotherapy in Patients With Completely Resected Stage IB Tumors Equal to or Larger Than 4cm) to Stage IIIA Anaplastic Lymphoma Kinase Positive Non-Small Cell Lung Cancer	ALK	IB-IIIA
NCT02576431	A Study to Learn How Well the Drug Larotrectinib Works in Adults With Different Solid Cancers With a Change in the Genes Called NTRK Fusion	NTRK1, NTRK2, or NTRK3 gene fusion	IIIB-IV

Indeed, intensified treatment approaches are often attractive for young patients, as an improved response in patients with limited or oligometastatic disease could allow local consolidative approaches with surgery and radiation to further improve survival outcomes. Intensification approaches unsurprisingly are associated with higher toxicity and treatment selection should be tailored to patient preferences and characteristics. Though osimertinib itself does not show pharmacokinetic differences between age or sex (116), the addition of chemotherapy unsurprisingly yields higher toxicities among age groups, which must be accounted for (113).

Lorlatinib as first line treatment for ALK-rearranged lung cancer has also shown impressive 5-year outcomes based on phase 3 CROWN study results, with median PFS and median time to intracranial progression still not reached, and a 5 year PFS rate of 60% (117). Patients under 65 years old accounted for 65% (n=193) of the total population and had an improved HR compared to their older counterparts (HR 0.22 vs 0.35). The central nervous system (CNS) protective role of lorlatinib makes it an attractive choice, especially given that young patients are more prone to brain metastases and disease progression in the CNS after treatment initiation, though toxicity often necessitates dose reduction at the outset of treatment. Compared with other ALK and ROS1 inhibitors, lorlatinib is associated with a unique set of spectrum of treatment-related adverse events (trAEs), including hyperlipidemia, peripheral neuropathy, and neurocognitive effects (118) that may render younger patients as being reluctant to begin lorlatinib in the first-line setting, despite its remarkable trial results. In the updated phase 3 CROWN study, grade 3–5 trAEs in the lorlatinib arm occurred in 67% of patients, of which 39%, 21%, and 5% led to temporary drug discontinuation, dose reduction, and permanent drug discontinuation, respectively. Though the majority (86%) of all-causality CNS AEs in the lorlatinib group were grade 1 or 2, and only three patients who experienced CNS trAEs permanently discontinued lorlatinib, CNS AEs occurred in six of nine (67%) of patients with prior brain radiotherapy and in 57 of 140 (41%) patients without prior brain radiotherapy. One recent study (118) of 144 patients with advanced ALK- or ROS1-rearranged NSCLC treated with lorlatinib in the second-line setting or later, too, found that 40% of patients had trAE- related dose reductions, most (59%) owing to neurocognitive AEs or neuropathy.

The TRIDENT-1 trial also showed robust efficacy of repotrectinib in patients with advanced ROS1 rearranged lung cancer, with an overall response rate (ORR) of 79%, median duration of response 34.1 months, and median PFS 35.7 months. The majority of TKI-naïve patients in this trial were 18–65 years old (n=52, 73%) and had an ORR of 81%, compared to the 65-75-year-old patients (n=15, 21%) with ORR 67% (95). In sum, though drugs with enhanced CNS penetration are more likely to be tolerated by young patients, these regimens are often accompanied by a unique set of adverse events, including cognitive changes and mood disorders that that may significantly impact a patient’s quality of life (QOL). Healthcare providers should counsel patients on expectations and consider utilizing a cognitive assessment tool or referral to a psychiatrist or therapist, if warranted (119).

Other more rare oncogenic drivers, such as NRG1 fusions, also recently received FDA approval for the bispecific antibody zenocutuzumab (120). Though this fusion is only found in approximately 0.2% of lung cancers (121), the global eNRGy1 Registry primarily observed its prevalence among individuals without a history of tobacco use (57%) (122), potentially offering an attractive opportunity to explore this fusion’s drug tolerability and clinical outcomes in the context of young patients.

For patients with no actionable mutations, the standard of care in the frontline for metastatic NSCLC includes the combination of chemotherapy and immunotherapy. Several landmark trials assess immunotherapy efficacy with or without chemotherapy in the metastatic setting. Some show a similar degree of benefit for patients across age groups (123–126), with no benefit for younger patients (127, 128), while others have demonstrated a more meaningful benefit in those <65 years (129–136). Immunotherapy is also being incorporated in the perioperative management of resectable NSCLC without detected alterations in EGFR or ALK and have also shown a similar degree of benefit, irrespective of age (137–139).

As previously mentioned, tumors with targetable driver alterations are often PD-L1 positive, likely due to the oncogene-driven activation of downstream signaling effectors and not true T cell-mediated immunogenicity that is typically associated with ICI responsiveness. Therefore, though young patients may derive less benefit from ICIs, and an increased rate of immune-related adverse events (irAEs) in adults 65 years or older (42%) compared to 18–64 year olds (33%) has been observed (140–142), the risks of irAEs for young patients still remain an important consideration (143). Early identification and mitigation of these AEs is particularly important given that young patients are more likely to experience the psychosocial and stress-related effects of therapy (144), as well as long-term AEs (99). Fatigue is commonly reported amongst young patients, with potential to significantly affect long term QOL (145); endocrine toxicity has also been observed to be more common in those under age 65 (142). Our analysis of 231 patients with NSCLC (146) found that women, specifically, were more likely to experience irAEs compared with men, but also significantly more likely to develop endocrinopathies (i.e. hypothyroidism, hypophysitis, type 1 diabetes). As these adverse events tend to have long-term repercussions on fertility and QOL, clinicians must therefore adequately caution and counsel young patients on realistic and lifelong irAE management strategies.

Lastly, despite the remarkable outcomes of precision medicine, attention must situate on how the overall length of life of young patients is still considerably lower than older patients diagnosed at an advanced age. A 2022 review (147) of 177 patients with advanced EGFR or ALK-positive NSCLC who received their first chemotherapy between December 2008- September 2015 found the median OS to be 40.6 months, the three-year survival rate to be 54%, and the five-year survival rate to be 28%. For patients with EGFRm-NSCLC, the median OS was 36.9 months, and for those with ALK-altered disease, the median OS was 55.4 months. Similarly, a 2025 cohort study (148) of 1,323 patients treated with first-line osimertinib found that while 58% of patients survived to 2 years, only 18% survived to 5 years. Even in the remarkable case that a 40-year-old individual with lung cancer survived for a decade, they still would have hardly reached midlife. For these patients, it is not solely the number of life years that is lost, but all that comes with it: pursuing passions, achieving goals, developing relationships, witnessing children’s milestones, advancing within work, and configuring hopes and dreams for the future (149).

## 5 Psychosocial needs

Young patients with lung cancer are diagnosed at a more disruptive time in their lives than their older counterparts, rendering them with distinct psychosocial needs. These young patients experience higher rates of emotional distress compared to older patients; with over 60% reporting significant distress (150). Though younger age is a strong predictor of heightened emotional challenges, patients with lung cancer in general report higher rates of depression and anxiety than those with other cancers (150–153), perhaps due to the chronic pain and fatigue conferred by the disease, which exacerbate emotional issues (150). Young patients may also be more apt to experience feelings of isolation (154, 155) due to not having peers facing similar challenges and not identifying with older patients whom they may meet throughout their cancer journey. Additionally, younger patients often face greater disease-related stigma (156), leading to feelings of guilt, shame (157), depression, anxiety (158), poorer QOL (159), and higher symptom severity (158); stigma may relate to diagnostic delays, limited use of adjunctive treatment and psychosocial support services, and low clinical trial enrollment (160). Young patients also experience a greater burden of familial responsibilities, as they are more likely to have dependent children and act as caregivers to parents. Further, with advancements in treatment, younger patients may live longer, but face ongoing fears of recurrence and long-term health impacts from their extensive cancer treatments (153). As younger patients are more likely to be on a greater number of lines of treatment, more research is warranted to explore the degree of distress engendered by these additional and/or more aggressive therapies.

Early and accurate identification of emotional issues, achieved through routine administration of validated tools to detect emotional distress, is crucial (161, 162). Healthcare providers should also be vigilant for changes in mood, withdrawal, or expressions of hopelessness, and encourage patients to openly share their feelings. Insights from family members or caregivers can also provide valuable perspectives. Counseling and psychotherapy (163) may also be helpful, particularly cognitive-behavioral therapy (CBT) interventions, which have shown efficacy in reducing anxiety and depression across various cancer populations (164, 165). Psychoeducational interventions that teach skills such as stress management may be particularly effective when combined with CBT (166, 167). Additionally, mindfulness-based therapies such as meditation and yoga have demonstrated moderate effects in reducing symptoms of depression and anxiety in patients with cancer (168–170) and may resonate well with younger individuals. As social connectedness has been found to mediate the relationship between social isolation and depression among young adult cancer survivors (155), facilitating peer connections through in-person or online support groups tailored for younger patients with cancer may also confer a shared identity and help combat feelings of isolation, with age-specific groups allowing for the sharing of unique concerns (171–173).

**Figure 2:**
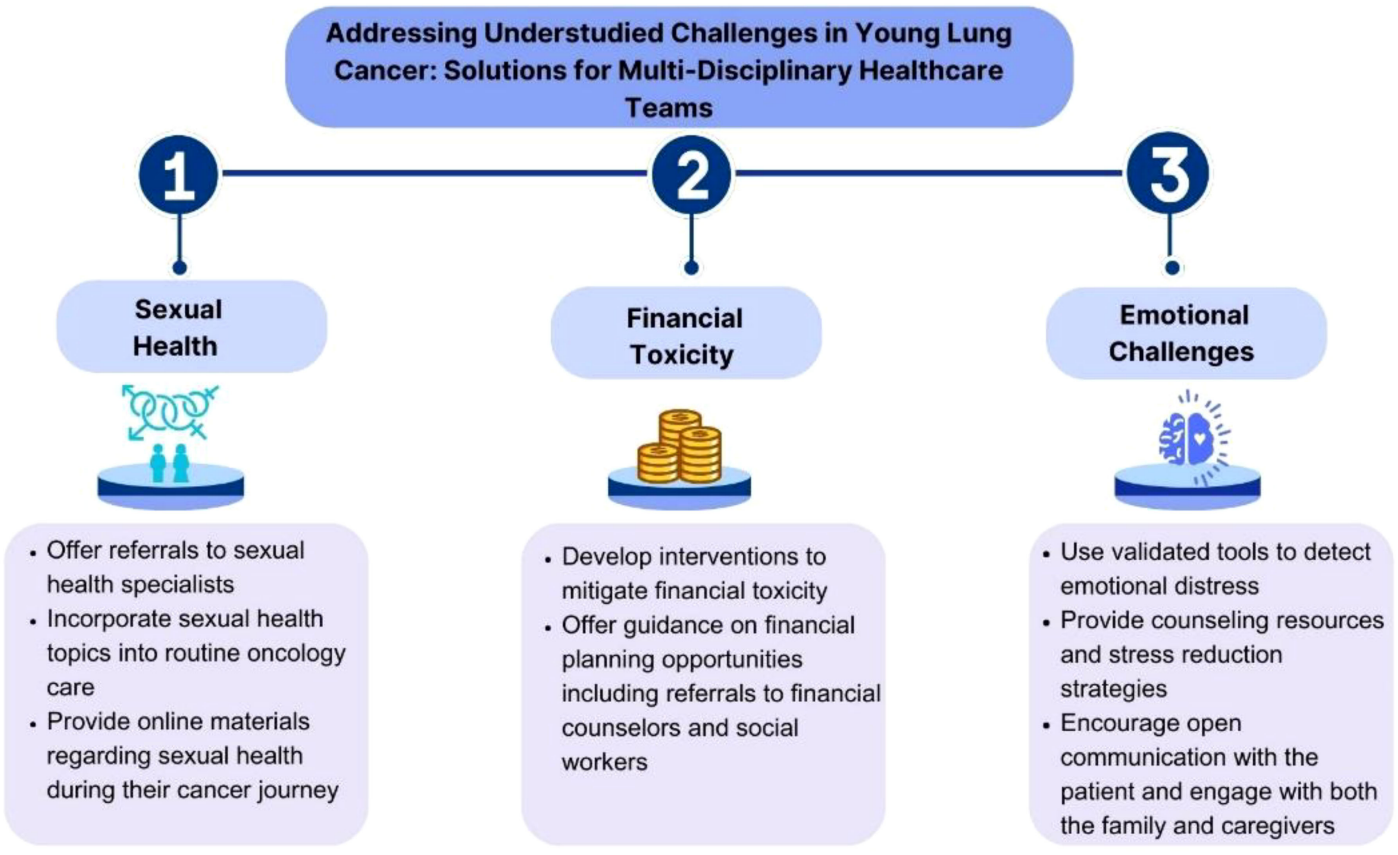
Multidisciplinary management of young lung cancer.

Financial toxicity is another significant concern for younger patients, who are often required to continue working for insurance coverage and support children or parents, exacerbating stress and limiting their ability to focus on treatment and recovery (174). More research is needed to identify the unique needs and most effective support for this growing population. The Young Lung Cancer: Psychosocial Needs Assessment at DFCI (174) is the first study to explore the psychosocial needs of young patients with lung cancer; preliminary results revealed how these patients report ample financial toxicity and issues impacting physical, emotional, and functional well-being. Specifically, a majority reported feeling sad or anxious (53%, 59%), and 57% were worried about premature death. Of a maximum score of 44, the mean COST score was 24, consistent with high financial toxicity in this population. Therefore, integrating the psychosocial support strategies into routine care and National Cancer Control Plans is essential for delivering comprehensive cancer care (**Figure 2**).

## 6 Fertility, pregnancy, and sexual health

The enhanced overall survival of young patients makes it important to elucidate the effects of treatments on fertility and sexual health. However, most contemporary knowledge has been derived from research conducted in animal models, from data extrapolated from patients with breast cancer (175) (a hormonal dependent cancer with differing clinic-pathologic characteristics), *and* before the incorporation of novel therapies such as ICIs and TKIs, which young patients are more likely to be on.

A global regulatory filings and public assessment analysis found that for female patients, 58% of ICI treatments represent a fertility risk, compared to only 33% for male patients (176). Animal studies of ICIs have also yielded unfavorable results including diminished ovarian follicular reserve, low maternal weight, excessive rates of abortion (177, 178), stillbirth, and premature delivery (177), compounded with negative long-term consequences such as hypogonadism, hypophysitis, and hypothyroidism (178). Similarly, early-generation TKIs have been found to decrease women’s total follicle count, negatively affect oocyte retrieval, ovarian reserve, reduce embryonic developmental potential, and produce teratogenic effects (179), though data regarding the effects of contemporary TKIs on oocytes and sex hormones is non-existant (180, 181).

A total of 66 cases of lung cancer diagnoses during pregnancy have been reported in the literature. Given the bleak and heterogenous aforementioned therapeutic effects on fertility, it is unsurprising that lung cancer treatment management throughout pregnancy is far from standardized. Though cytotoxic therapy has been commonly used throughout pregnancy, chemotherapy is typically avoided during the first trimester and instead given throughout the second and third trimesters, as risks include spontaneous abortion, congenital abnormalities, and transplacental transmission of taxanes. While some cases report safe TKI use, other cases have ended in spontaneous abortion or resulted in fetal growth restriction, treatment-related malformations, and severe maternal adverse effects. Surgery may be considered in highly specific cases of early-stage lung cancer or as a palliative measure for patients with extensive bone metastasis or those at risk for spinal cord compression. While novel radiation techniques have reduced toxicity to surrounding healthy tissue, routine radiation is not recommended, as some radiation still reaches the fetus. Regarding imaging, PET/MRI is preferred, though doses as low as reasonably possible should still be applied; ultrasound techniques can also be used to evaluate pleural effusions, mediastinal staging, and liver involvement (182). To aid in the development of a systematic and data-based understanding of how to treat pregnant young women with lung cancer, researchers at DFCI launched the International Pregnancy and Lung Cancer Registry to continuously learn about the impact of lung cancer treatment on maternal and fetal outcomes. An initial review of the first 22 cases was recently presented at the 2024 World Conference on Lung Cancer (183); results demonstrated a lack of uniformity in treatments utilized, treatment deferrals, and imaging modalities, highlighting the need for an increased and data-driven understanding to optimally support this vulnerable population of young patients.

Sexual health is another important consideration, as the health effects of sexual activity on psychological wellness have been widely documented (184, 185) and ensuring that young people experience good sexual health is a key public health concern (186, 187). Among a nationally representative sample of U.S. adults (188), high importance of sexual health to QOL was reported by 62% of men and 43% of women, particularly those in their mid-30s to mid-40s. Sexual dysfunction has been related to higher symptom distress (189) and worse functional status in patients with lung cancer, and among young adults with cancer, sexual dysfunction amplifies feelings of isolation and emotional distress (190–193). In our Sexual Health Assessment for Women with Lung Cancer (SHAWL) Study (194), nearly half of patients reported minimal satisfaction with their sex life. The majority (74%) of young patients also indicated having minimal interest in sexual activity, emphasizing the need to integrate sexual health counseling within routine oncology care for young patients with lung cancer.

Despite the importance, oncologists have suboptimal knowledge, practices, and attitudes on fertility preservation and sexual health throughout cancer treatments (14–16). Young women are rarely adequately counseled regarding options for future fertility (195), unlike men, who are frequently advised to preserve sperm. Women have expressed negative sentiments regarding fertility preservation, citing inadequate information and presentation of available options as contributing factors (196, 197). Similarly, in a survey of 120 medical oncologists, 81.5% reported that they discussed sexual function with fewer than half of patients, citing a lack of training as primary cause. Social media is a largely untapped resource that can be used to disseminate accessible information about sexuality to young patients. In addition to offering referrals to sexual health specialists, open-ended discussion frameworks may also serve as a helpful origin source for oncologists to incorporate inclusive sexuality care into their practice (198).

Women, especially those belonging to medically underserved populations such as racial and ethnic minorities, rural residents, or those who are low-income or uninsured, are also disproportionately impacted (199) by the costly and invasive nature of fertility preservation (99), which typically involves hormone stimulation, ultrasounds, bloodwork, and surgical procedures. Ovarian stimulation and egg retrieval cycle may take up to 14 days if successful, potentially delaying the initiation of cancer treatment (200). In contrast, male fertility tends to be a non-invasive, cost-effective, timely, and efficient process. Many insurance plans also do not cover procedural costs, adding to the existing financial strain of cancer treatments. While sperm preservation can cost several hundred dollars, oocyte or embryo cryopreservation can range from $6,000 to $15,000 per cycle, excluding medication and storage fees. A recent study also revealed significant geographic disparities in access to oncology fertility preservation care, only exacerbating the aforementioned disparities (201).

## 7 Palliative care and end-of-life care

Early integration of palliative care is crucial in the comprehensive management of young patients, aiming to improve their emotional symptoms, QOL, and OS (202). Systematic reviews emphasize the significance of culturally sensitive approaches in end-of-life (EOL) care, respecting patient preferences, maintaining human dignity, and facilitating open communication within family dynamics (203, 204). Research comparing early virtual palliative care versus in-person visits has shown equivalent effects on QOL in patients with advanced NSCLC, highlighting the feasibility and effectiveness of telemedicine in providing supportive care (205). Additionally, stepped-care model, associated with fewer days in hospice, offers a scalable approach to delivering early palliative care and enhancing patient-reported outcomes (206). Despite these advancements, biases against supportive care persist, often resulting in young patients to be referred to hospice only when it is too late to benefit.

**Figure 3:**
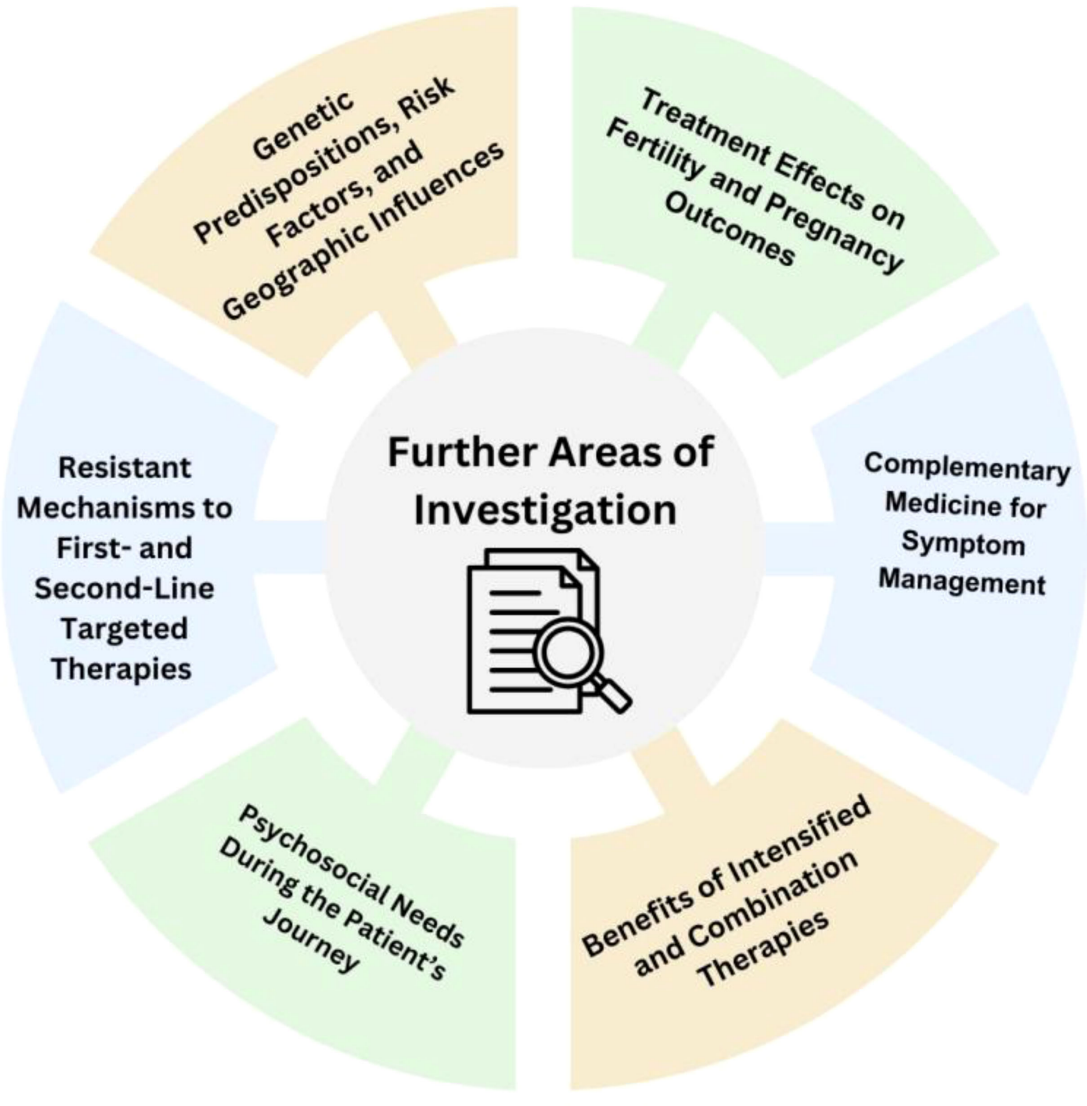
Future areas of investigation for young lung cancer.

Complementary medicine, including cannabis use, has also emerged as a potential adjunctive therapy in managing symptoms and improving QOL for patients with advanced cancer (207). However, ASCO guidelines suggest that evidence still remains insufficient to recommend for or against cannabis in managing cancer treatment-related symptoms, except for alleviating treatment-related nausea or vomiting with dronabinol, nabilone, or a 1:1 THC extract (207) (**Figure 3**). Indeed, a recent umbrella review (208) of global epidemiological evidence on the cancer risk of cannabis use based upon publications since 2017 found moderate evidence of no statistical association between cannabis smoking and the incidence of lung cancer. However, difficulties remain in quantifying and soliciting data on cannabis use, especially in the context of confounding factors such as tobacco exposure, emphasizing the importance of future research that incorporates cannabis use history when reporting on disease outcomes. Though clinicians are encouraged to inquire about cannabis use, educate patients on risks, and minimize potential harm (207), recommendations specific to young patients with lung cancer are underexplored.

Though evidence has found advance care planning (ACP) to be well-liked among young adults and improve caregiver decisional certainty, ACP among young patients with cancer remains clinically underutilized (209). Advanced directives (AD), a component of ACP, contributes to the attenuation of patient suffering and anxiety, psychological relief and patient satisfaction (210), though AD may be difficult to introduce among young patients. Due in part to the lack of standards to guide the quality, content, approach, and timing of ACP discussions, as well as physician belief that EOL discussions will lead patients to higher levels of stress and discomfort (210), late EOL discussions and completion of AD often occurs during the last 3 months of life or later (211), when aggressive care preferences (associated with worse QOL and worse bereavement adjustment) are more likely (212). Promoting the completion of AD early in the disease trajectory will empower young patients to articulate their care preferences, ensuring alignment with their values during critical moments.

At the moment of death, though optimizing pain management to promote comfort and dignity is crucial, addressing psychological needs through facilitating family presence and providing spiritual and emotional support to both patient and caregivers is also necessary. Early integration of palliative care in the disease trajectory will allow for the comfortable integration of these measures to meet patients’ needs with sensitivity and understanding.

## 8 Conclusions

Young patients with lung cancer represent a distinct population, with unique disease and treatment-related characteristics and psychosocial and survivorship needs. Young patients with lung cancer are more likely to harbor a targetable oncogenic driver and be diagnosed with advanced disease, highlighting the importance of disease awareness, unbiased attention to symptoms, and early biomarker testing. However, further research is needed to explore the influence of genetic predisposition, comorbidities, geographic location, and other risk factors in the development of lung cancer in young adults. Clinical trials are also crucial to guide evidence-based decisions on therapy selection and customization, the incorporation of targeted therapies, and escalation and de-escalation strategies.

Young patients with lung cancer also demonstrate improved survival, underscoring the need for comprehensive research efforts that evaluate disease-related effects on survivorship needs such as fertility, financial toxicity, and psychosocial well-being. Healthcare providers should screen for distress and implement an integrated approach combining psychotherapies like CBT, mindfulness training, peer support, and targeted psychoeducation, to provide tailored psychological support, combat stigma, and support family dynamics. Training in palliative care and promoting a positive attitude towards supportive care is also essential. In this way, comprehensive cancer care can be tailored to this unique yet understudied patient population.
